# Arsenic‐Containing Phosphatidylcholines: A New Group of Arsenolipids Discovered in Herring Caviar

**DOI:** 10.1002/ange.201512031

**Published:** 2016-03-21

**Authors:** Sandra A. Viczek, Kenneth B. Jensen, Kevin A. Francesconi

**Affiliations:** ^1^ Institute of Chemistry: Analytical ChemistryNAWI GrazUniversity of GrazUniversitätsplatz 18010GrazAustria

**Keywords:** Arsen, Arsenolipide, Phosphatidylcholine, Phospholipide

## Abstract

A new group of arsenolipids based on cell‐membrane phosphatidylcholines has been discovered in herring caviar (fish roe). A combination of HPLC with elemental and molecular mass spectrometry was used to identify five arsenic‐containing phosphatidylcholines; the same technique applied to salmon caviar identified an arsenic‐containing phosphatidylethanolamine. The arsenic group in these membrane lipids might impart particular properties to the molecules not displayed by their non‐arsenic analogues. Additionally, the new compounds have human health implications according to recent results showing high cytotoxicity for some arsenolipids.

## Graphical abstract

**Eine komplexe Delikatesse**: Arsenhaltige Phosphatidylcholine und ein arsenhaltiges Phosphatidylethanolamin wurden in Fischrogen identifiziert. Die Einführung einer Arsengruppe in typische Membranlipide könnte den Molekülen Eigenschaften verleihen, die bei arsenfreien Verbindungen nicht vorkommen.

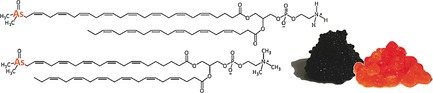

Arsenic‐containing lipids, so‐called arsenolipids, are natural constituents of marine organisms thought to be involved in arsenic detoxification processes.^1^, ^2^ The first arsenolipid to be rigorously identified was an arsenosugar lipid which was isolated from a brown alga.^3^ This lipid was related to the major water‐soluble arsenic compounds found in marine algae,^4^ and seemed to provide a plausible rationale for the biosynthesis of these lipids as part of an arsenic detoxification process.^2^, ^5^

Research activity over the last eight years, however, has revealed several new groups of naturally occurring arsenolipids, including arsenic‐containing fatty acids^6^, ^7^ (AsFA) and hydrocarbons^8^, ^9^ (AsHC; Figure 1), which clearly have a biosynthetic origin different from that of the arsenosugar lipids. The widespread distribution of these arsenolipids among marine organisms, including those used as human food, encouraged the chemical synthesis of the compounds^10^ and investigations of their toxicological properties.^11^, ^12^ A more fundamental biological role for the compounds, for example in membrane chemistry, has also been suggested,^6^ but so far there is no evidence supporting this speculation.

**Figure 1:**
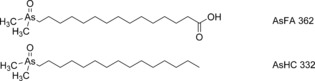
Examples of two major, known groups of arsenolipids: AsFA=arsenic‐containing fatty acid; AsHC=arsenic‐containing hydrocarbon. The number after the abbreviation is the nominal molecular mass of the displayed example.

A recent study of a fish oil showed the presence of an unidentified group of arsenolipids thought to be conjugates of arsenic fatty acids because they could be base‐hydrolyzed to known arsenic fatty acids.^13^ We wondered if this group of arsenolipids might be related to phosphatidyl compounds integral to membrane chemistry. But in the fish oil samples, the fatty acid conjugates were present at such trace levels that they could not be identified. Seeking a richer source of cell membrane compounds, we turned to fish eggs (roe).^14^ Herein, we report the identification of a major new group of arsenolipids from three samples of herring roe (herring caviar) and a preliminary investigation of arsenolipids in salmon caviar.

Extracting freeze‐dried herring caviar (*Clupea harengus*; ≈0.8 μg As g^−1^ dry mass, originating from the Norwegian Sea) with DCM/MeOH and washing the extract with water yielded an organic layer containing approximately 80 % of the initial arsenic. After all traces of DCM were removed, the residue was re‐dissolved in absolute ethanol and analyzed by reversed‐phase HPLC coupled to an elemental mass spectrometer (inductively coupled plasma mass spectrometry, ICPMS). The HPLC/ICPMS measurements performed in selected‐ion monitoring mode with *m*/*z* 75 (As^+^) indicated the presence of several known arsenic fatty acids and arsenic hydrocarbons, in addition to a cluster of later‐eluting arsenolipids (RT 25–28 min; Figure 2 a). Silica column chromatography, previously used to purify known arsenolipids,^15^ was found to be ineffective for this group of arsenolipids, possibly because it degraded the native compounds. Thus, further mass spectrometric analyses were performed on the crude extracts by using HPLC coupled via electrospray ionization (positive mode) to a high‐accuracy mass spectrometer with collision‐induced‐dissociation capability (Figure 2 b). During the elution, a large number (approximately 700) of distinct ions were isolated and fragmented, yielding more than 18 000 MS/MS spectra in each run (Supporting Information).

**Figure 2:**
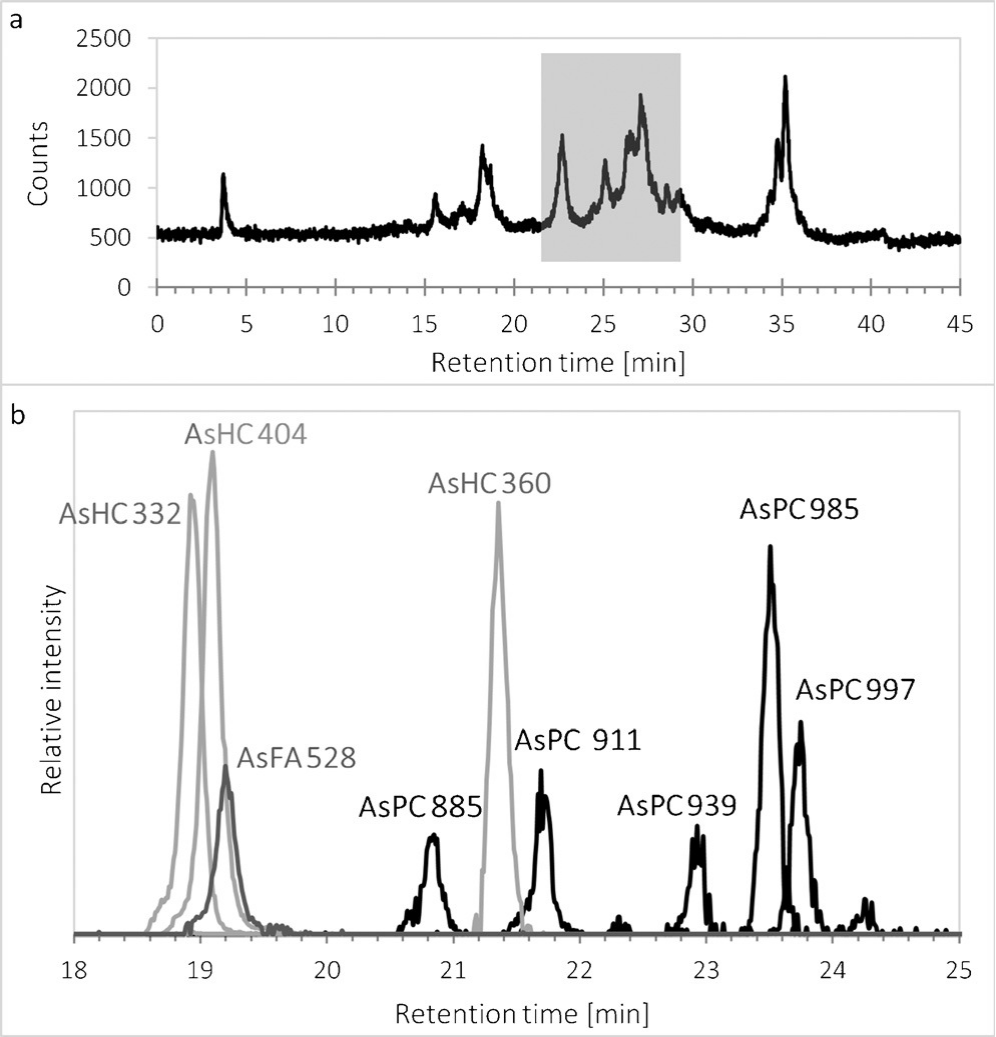
a) HPLC/ICPMS chromatogram, and b) HPLC/ESIMS extracted ion chromatogram of a lipid extract from herring caviar. Small differences in the HPLC measurement conditions resulted in a uniform shift in retention times between the two measurements. AsHC 332, for which we had an authentic standard,^10^ could be used to normalize the two HPLC data sets (RT=22.5 min by HPLC/ICPMS and RT=18.9 min by HPLC/ESIMS. See Figure 1 and Figure 5 for peak/compound designations.

Almost all of the arsenolipids identified so far contain the dimethylarsinoyl group −As(O)Me_2_, which gives rise to two characteristic fragment ions, one with *m*/*z*=105 (C_2_H_6_As^+^) and one with *m*/*z*=123 (C_2_H_8_OAs^+^), when subjected to collision‐induced‐dissociation. Owing to their mass defects, these fragment ions can be uniquely identified by a high‐resolution mass spectrometer even from a complex matrix. Searching the fragmentation spectra for these two fragment ions exposed a multitude of precursor ions, from which six known arsenic fatty acids (AsFA 334, AsFA 362, AsFA 388, AsFA 390, AsFA 436, AsFA 448) and five known arsenic hydrocarbons (AsHC 330, AsHC 332, AsHC 358, AsHC 360, AsHC 404) could be expressly identified (Supporting Information, Table S2 and Figure S2). This procedure similarly implied an ion with [*M*+H]^+^ at *m*/*z=*529. The accurate mass and fragmentation data of this ion were consistent with a hitherto unknown arsenic fatty acid with eight ethylene groups (AsFA 528; Figures 3 and S3).

**Figure 3:**
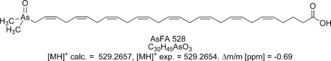
Proposed structure of the new fatty acid AsFA 528. Position and geometry of double bonds have not been determined.

In the later part of the HPLC/ESIMS chromatogram (RT 20.5–24 min; Figure 2 b), weak signals for one or the other of the two indicator fragments were observed. The precursor ions were singly or doubly charged, and had masses revealing the presence of an odd number of nitrogen atoms in their formulae. Also present were fragment ions attributable to a previously observed arsenic‐containing (free) fatty acid and its ketene derivative. Five such examples were discovered. The observed masses were all consistent with the general formula C_*x*_H_*y*_O_9_NAsP, and inspection of the MS/MS (fragmentation) spectra of these ions confirmed similarity as well as a set of prominent fragment ions characteristically observed in the MS/MS spectra of phosphatidylcholines.^16^ An example is given in Figure 4. These observations indicated the presence of phosphatidylcholine compounds where one of the fatty acids is an arsenic fatty acid. In phosphatidylcholines, fragment ions relating to the fatty acid moieties are normally of minor intensity.^16^ In contrast, for the arsenic‐containing phosphatidylcholines (AsPCs), two significant ions representing the protonated free arsenic fatty acid and the protonated ketene can be seen. This difference is an expected consequence of the proton affinity of the arsinoyl group of the As fatty acids. Based on these data, five AsPCs were identified (Figure 5 and Table 1). It should be noted that, while the phosphatidylcholine‐revealing fragment ions are conspicuous, the small arsenic‐containing fragments that led to the discovery of these ions are not.

**Figure 4:**
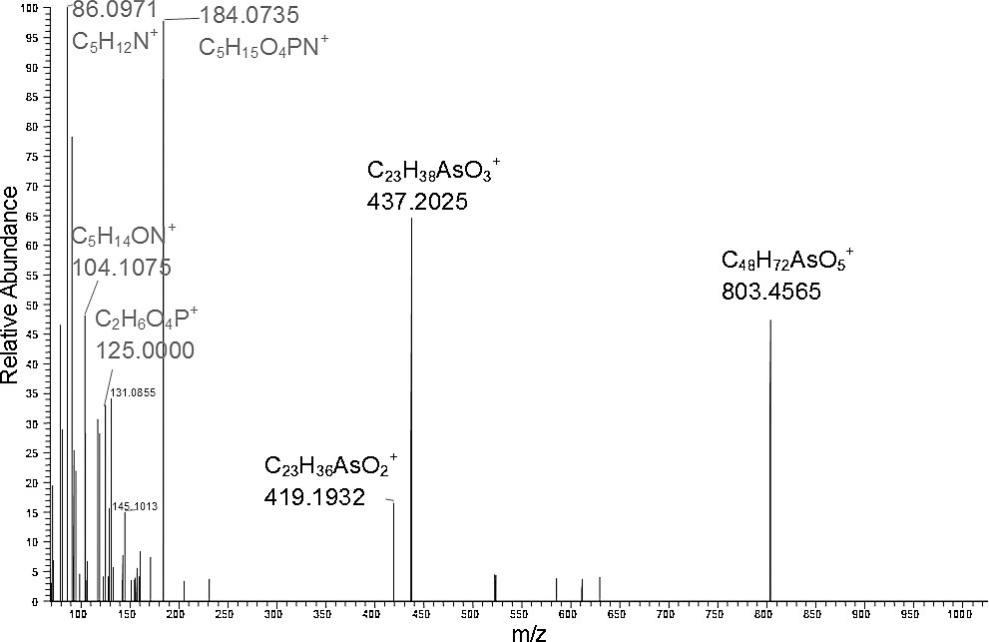
Fragmentation of the singly charged ion with *m*/*z*=986 (AsPC 985). A similar pattern was displayed by the other four AsPCs in the lipid extracts from herring caviar (Supporting Information, Figures S4–S8).

**Figure 5:**
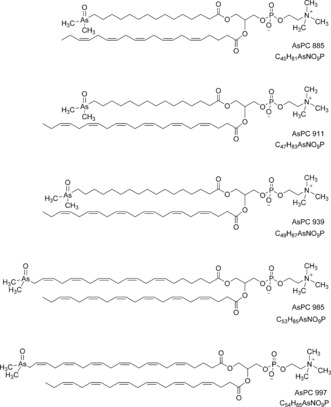
Proposed structures for the five AsPCs found in herring caviar. The compounds are displayed as one of the two possible glycerol ester positional isomers. Position and geometry of double bonds in the fatty acids have not been determined. The four arsenic fatty acids (AsFA 362, AsFA 390, AsFA 436, AsFA 448) contained in the five AsPCs were the same as those identified also in unbound form in the herring caviar.

**Table 1:** Singly and doubly charged masses of five AsPCs and AsPE 1035.

	[*M*+H]^+^ [*M*+2H]^2+^ exp.	[*M*+H]^+^ [*M*+2H]^2+^ calc.	|Δm/m| [ppm]	Formula of neutral species
AsPC 885	886.4950 443.7507	886.4937 443.7505	1.4 0.3	C_45_H_81_O_9_NAsP
AsPC 911	912.5091 456.7580	912.5094 456.7583	0.4 0.7	C_47_H_83_O_9_NAsP
AsPC 939	940.5429 470.7737	940.5407 470.7740	2.3 0.5	C_49_H_87_O_9_NAsP
AsPC 985	986.5248 493.7661	986.5250 493.7661	0.2 <0.1	C_53_H_85_O_9_NAsP
AsPC 997	998.5253 499.7660	998.5250 499.7661	0.2 0.3	C_54_H_85_O_9_NAsP
AsPE 1035	1036.5387 518.7728	1036.5413 518.7740	1.9 2.3	C_57_H_87_O_9_NAsP

The collision energy used was originally optimized to obtain intense arsinoyl‐group fragments, and therefore was too high to partly preserve the phosphatidylcholine precursor ions when fragmenting these. Consequently, in Figure 4, the precursor ion at *m*/*z*=986.5250 is absent. In contrast, a prominent fragment ion at *m*/*z*=803 (corresponding to a re‐arranged diacylglycerol‐type fragment resulting from the loss of the neutral phosphorylcholine zwitterion^16^) and protonated phosphorylcholine at *m*/*z*=184, along with the smaller assigned fragments at *m*/*z=*86, 104, and 125, are present together with the two above‐mentioned fragment ions of the arsenic fatty acid ester. An analogous fragmentation pattern was observed for the other four AsPCs (Table 2).

**Table 2:** Main fragments of AsPCs, experimental [*M*+H]^+^ masses and |Δm/m| values (ppm).

	AsPC 885	AsPC 911	AsPC 939	AsPC 985	AsPC 997
86.0970 C_5_H_12_N^+^	86.0971 (7.8)	86.0971 (7.9)	86.0970 (6.8)	86.0971 (7.4)	86.0972 (8.4)
					
104.1064 C_5_H_14_ON^+^	104.1072 (2.0)	104.1075 (5.3)	104.1073 (2.7)	104.1075 (4.7)	104.1076 (5.6)
					
124.9993 C_2_H_6_O_4_P^+^	125.0000 (1.6)	125.0000 (0.9)	125.0000 (1.6)	125.0000 (1.2)	125.0000 (1.7)
					
184.0738 C_5_H_15_O_4_N^+^P	184.0735 (0.8)	184.0734 (0.2)	184.0733 (0.1)	184.0735 (1.0)	184.0735 (1.0)
					
AsFA [*M*+H]^+^	363.1873 (0.7)	363.1870 (1.4)	391.2181 (1.7)	437.2025 (1.5)	449.2027 (0.9)
					
AsFA [*M*+H]^+^ −18.0106	345.1764 (1.5)	345.1769 (<0.1)	373.2075 (1.9)	419.1932 (1.4)	431.1934 (1.8)
					
[*M*+H]^+^ −183.0660^[a]^	–^[b]^	–^[b]^	757.4713 (4.5)	803.4565 (3.2)	815.4631 (5.0)

[a] Mainly in MS/MS spectra of singly charged species. [b] No MS/MS spectrum of singly charged species obtained.

Phosphatidylcholines form a major lipid class comprising many compounds varying only in their fatty acid composition. It is thus likely that the cluster of unresolved arsenic compounds with HPLC/ICPMS retention times 25–28 (Figure 2 a), which collectively constitute roughly 50 % of the total arsenic, belong predominantly to the new group of arsenic phosphatidylcholines described here. This study identified five of those compounds, and opens the door to discovering many more of these complex arsenic natural products.

Examination of two other sources of herring caviar purchased in Sweden produced very similar patterns of arsenolipids (Table S2), and both contained the five AsPCs described above. The same approach applied to an extract of salmon caviar (*Oncorhynchus tshawytscha*; ≈0.7 μg As g^−1^ dry mass, originating from Alaska) did not reveal the presence of AsPCs, but again AsFA 528 ([*M*+H]^+^ exp.: 529.2642; |Δm/m| [ppm]: 2.9) and also an arsenic‐containing phosphatidylethanolamine (AsPE) incorporating AsFA 528 were identified (Figure 6 and Table 1). Arsenic‐containing phosphatidylethanolamines were not found in herring caviar, but as for the AsPCs in salmon caviar, we cannot exclude their presence at low levels (which indeed appears likely).

**Figure 6:**
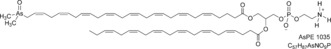
Proposed structure of AsPE 1035 found in salmon caviar. Position and geometry of double bonds in the fatty acid residues and their glycerol ester positions have not been determined.

The fragmentation behavior of the protonated AsPE 1035 (Figure S19) parallels the behavior observed for the protonated AsPCs with the exception that protonated phosphorylethanolamine at *m*/*z*=142 is not present. Such a mass‐spectrometric difference has also been noted between usual (non‐arsenic‐containing) PEs and PCs.^17^ The fragment ions of the arsenic fatty acid ester as well as the re‐arranged diacylglycerol‐type fragment (loss of the neutral phosphoryl‐ethanolamine zwitterion) were observed in the MS/MS spectrum of the singly charged species. In addition, the MS/MS spectrum of the doubly charged species revealed a fragment ion at *m*/*z*=585.2919, corresponding to the arsenic fatty acid with part of the glycerol molecule after loss of phosphorylethanolamine and docosahexaenoic acid as the corresponding ketene.

The origin of the arsenic phosphatidylcholines in the membrane‐rich fish eggs remains an open question. Phosphatidylcholines are key components of biological membranes. It is possible that infidelity in their biosynthesis results in the arsenic analogues, although a more fundamental role for the arsenic compounds cannot be dismissed. These compounds are likely to be widespread in fatty fish, based on the ubiquity of free arsenic fatty acids and the recent report of unidentified arsenic fatty acid conjugates in fish oil,^13^ and thus they have relevance to human health. Preliminary toxicity testing with human cells has shown that one group of arsenolipids, the arsenic hydrocarbons, are highly cytotoxic, and that their enhanced toxicity relative to arsenic fatty acids might be related to their less‐polar nature.^11^, ^12^ The arsenic phosphatidylcholines and the phosphatidylethanolamine reported here are, based on reversed‐phase HPLC retention times, less polar than the arsenic hydrocarbons, and thus might also be expected to be cytotoxic. In future work, we will synthesize representative members of this new group of arsenolipids to enable a full assessment of their chemical and toxicological properties.

## Supporting information

As a service to our authors and readers, this journal provides supporting information supplied by the authors. Such materials are peer reviewed and may be re‐organized for online delivery, but are not copy‐edited or typeset. Technical support issues arising from supporting information (other than missing files) should be addressed to the authors.

Supplementary
